# Indication of Horizontal DNA Gene Transfer by Extracellular Vesicles

**DOI:** 10.1371/journal.pone.0163665

**Published:** 2016-09-29

**Authors:** Stefanie Fischer, Kerstin Cornils, Thomas Speiseder, Anita Badbaran, Rudolph Reimer, Daniela Indenbirken, Adam Grundhoff, Bärbel Brunswig-Spickenheier, Malik Alawi, Claudia Lange

**Affiliations:** 1 Research Department Cell and Gene Therapy, Clinic for Stem Cell Transplantation, University Medical Center Hamburg-Eppendorf, Hamburg, Germany; 2 Research Unit Viral Transformation, Heinrich Pette Institute, Leibniz Institute for Experimental Virology, Hamburg, Germany; 3 Dept. Electron Microscopy, Heinrich Pette Institute, Leibniz Institute for Experimental Virology, Hamburg, Germany; 4 Research Group Virus Genomics, Heinrich Pette Institute, Leibniz Institute for Experimental Virology, Hamburg, Germany; 5 Bioinformatic Core, University Medical Center Hamburg-Eppendorf, Hamburg, Germany; Universita degli Studi di Torino, ITALY

## Abstract

The biological relevance of extracellular vesicles (EV) in intercellular communication has been well established. Thus far, proteins and RNA were described as main cargo. Here, we show that EV released from human bone marrow derived mesenchymal stromal cells (BM-hMSC) also carry high-molecular DNA in addition. Extensive EV characterization revealed this DNA mainly associated with the outer EV membrane and to a smaller degree also inside the EV. Our EV purification protocol secured that DNA is not derived from apoptotic or necrotic cells. To analyze the relevance of EV-associated DNA we lentivirally transduced *Arabidopsis thaliana*-DNA (*A*.*t*.-DNA) as indicator into BM-hMSC and generated EV. Using quantitative polymerase chain reaction (qPCR) techniques we detected high copy numbers of *A*.*t*.-DNA in EV. In recipient hMSC incubated with tagged EV for two weeks we identified *A*.*t*.-DNA transferred to recipient cells. Investigation of recipient cell DNA using quantitative PCR and verification of PCR-products by sequencing suggested stable integration of *A*.*t*.-DNA. In conclusion, for the first time our proof-of-principle experiments point to horizontal DNA transfer into recipient cells via EV. Based on our results we assume that eukaryotic cells are able to exchange genetic information in form of DNA extending the known cargo of EV by genomic DNA. This mechanism might be of relevance in cancer but also during cell evolution and development.

## Introduction

Mesenchymal stromal cells (MSC) have emerged as promising therapeutic tool for tissue regeneration. Secretion of bioactive molecules is now believed to be the main mechanism by which MSC achieve their therapeutic effect [1]. As suggested by studies in man and rodents, MSC provide trophic signals that inhibit apoptosis and fibrosis and stimulate angiogenesis and mitogenesis [2–4]. However, administered MSC did not home to the injured tissues but were trapped in the lungs after *i*.*v*. injection without long term survival [5]. Nonetheless, MSC showed the full range of protective properties. But how do MSC transfer their protective potential to the side of need?

In the late 1990s, Raposo *et al*. and Zitvogel *et al*. published the first reports about shed vesicles as important mediators of intercellular communication [6, 7]. Today it is assumed that most if not all cells actively release diverse types of membrane vesicles of endosomal and plasma membrane origin called exosomes and microvesicles into the extracellular environment, respectively. Here we will use the term extracellular vesicles (EV) to describe all classes of extracellular membrane vesicles with a size of 30 to 1,000 nm [8] since most methods purifying EV from *in vitro* and *in vivo* fluids detect a mixed population [8, 9]. EV are enclosed by phospholipid bilayers to transfer membrane and cytosolic proteins, lipids, and RNA and therefore might be less susceptible to degradation when leaving the cells of origin. This assumption is supported by detection of EV in various biological fluids, thus demonstrating their secretion *in vivo* and *in vitro* [10]. The observation that EV-associated mRNAs could be translated into functional proteins by target cells strongly influenced the research field [11, 12]. For example, epigenetic reprogramming of adult haematopoietic stem/progenitor cells by EV derived from murine embryonic stem cells and induction of angiogenesis mediated by endothelial cell-derived EV have been demonstrated [12, 13]. Additionally, EV bear either activating as well as inhibitory effects depending on the physiological state of the donor cells [9]. After all, not only healthy, but also neoplastic cells have been found to secrete EV [10, 14–20]. Tumor cell-derived EV might play a pivotal role in local and systemic cell-cell communication in cancer. For example, tumor-derived exosomes promote the formation of pre-metastatic niches through uptake of melanoma-derived exosomes by bone marrow-derived cells [14], exchange proteins with oncogenic activity, delivered to cells lacking this mutant form [21], contribute to multiple myeloma (MM) disease progression through release of MM bone marrow-MSC derived exosomes [22] and influence the invasive behaviour of breast cancer cells [15]. Conversely, EV/exosomes have been intensively investigated for their use in immunotherapy showing antitumor immune responses *in vivo* by using dendritic cell-derived exosomes [7, 23].

To this end, studies revealed that paracrine reparative functions of MSCs could, at least in part, be mediated by EV similarly to whole cells in kidney injury [24], myocardial ischemia [25] and lethal irradiation [26].

In our study, we detected high-molecular DNA in association with EV which were harvested from human bone marrow derived mesenchymal stromal cell (BM-hMSC) cultures and asked for the relevance of this finding. To our knowledge our proof of principle experiments indicate for the first time that EV-mediated horizontal DNA transfer reaches beyond the protein and RNA cargo and might be one of the mechanisms which creates cellular diversity in normal development, but also in cancer progression.

## Results

### Characterization of EV

**Fig 1:**
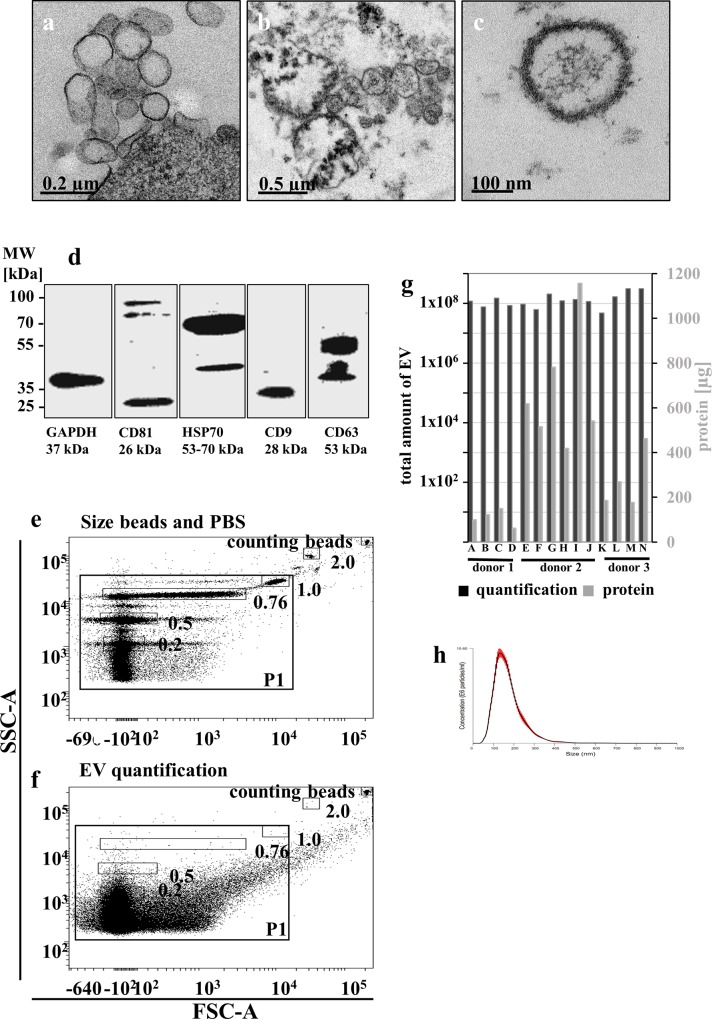
Characterization of EV. Human MSC were cultured on ibidi μ-slides, fixed and analyzed using electron microscopy. Shown is a part of cell membrane of a hMSC releasing EV (a). After ultracentrifugation of the supernatant, EV were resuspended in small volumes, sucked into carbo-tubes, fixed and analyzed using electron microscopy. Patches (b) and single EV (c) of 50–1,000 nm were detected. Ten μg of characteristic EV-proteins (CD81, HSP70, CD9 and CD63, GAPDH as housekeeper) were analyzed by Western blot (d). For quantification of EV using flow cytometry, size beads ranging from 0.2–2 μm were used to define the EV analysis area P1 (e) and impurities of 0.1μm filtered PBS in P1 (f). Purified EV in P1 were quantified using counting beads excluding the particles contained in filtered PBS. Total EV amounts per harvest (samples A-N from three individual donors) blotted against the protein content of each EV harvest revealed interindividual differences in protein cargo but reproducibility within one donor culture after repeated EV harvests (g). To investigate the underestimation of EV due to “swarm detection” in flow cytometry, 6 EV harvests were measured with NanoSight revealing ca. 1,000 fold higher concentration (401 ± 290) with a mode size of 146 ± 7.7 nm (h).

BM-hMSC and concentrated EV from hMSC supernatants were investigated using electron microscopy. Cells *in vitro* released EV with diameters of 50–150 nm from their plasma membrane (Fig 1A). At this point, we cannot decide whether exosomes or shedding microvesicles were detected. Importantly, cell cultures regularly displayed a viability of >97% as determined by Trypan-blue staining after harvest of supernatant for EV purification. After ultracentrifugation, concentrated EV consisted of a mixture of both vesicle specimens with diameters mainly < 0.8 μm (Fig 1B and 1C). EV displayed round structure in electron microscopy using our fixation protocol (Fig 1C). The characteristic proteins for exosomes/microvesicles CD81, HSP70, CD9, and CD63 have been detected via Western blot analysis (Fig 1D). Flow cytometry-based quantification of EV revealed that the major vesicle population was < 0.76 μm (Fig 1E: size beads and PBS particle contamination; Fig 1F: quantification of EV with counting beads) with a prominent population of ≤ 0.2 μm. Comparing protein quantification and EV counts for each separate harvest we observed interindividual variation of protein amounts between the different donor cultures but reproducible protein content within EV harvests from one defined donor culture (Fig 1G). Donor 1 (A-D) revealed to carry low amounts of protein, donor 2 (E-J) high protein amounts and donor 3 (K-N) intermediate protein amounts. Therefore, the number of EV used for horizontal transfer was calculated regarding to the number of producing cells.

Because of the “swarm detection” of small particles even with dedicated flow cytometry [27] we measured the absolute EV numbers employing NanoSight (Fig 1H). The underestimation in FACS represented a factor of approximately 1:1000 (401.292 ± 290.309, n = 6), demonstrating the FACS based quantification as a method for estimating the rough EV amounts when taking into account the established factor. The size of EV established in NanoSight (146 ± 7.7 nm) corresponds to the FACS data.

Altogether our results show constant size distribution of EV in electron microscopy, flow cytometry and NanoSight (Fig 1A–1C, 1F and 1H), uniform antigen expression of concentrated EV (Fig 1D), similar yield of EV from one donor-culture comparing the repeated harvests, and defined protein cargo for the respective donor hMSC derived EV-harvest (Fig 1G). The results gained for *A*.*t*.-transduced hMSC-derived EV were similar to those from non-transduced hMSC-derived EV (S1A and S1B Fig).

### Detection of DNA from EV

**Fig 2:**
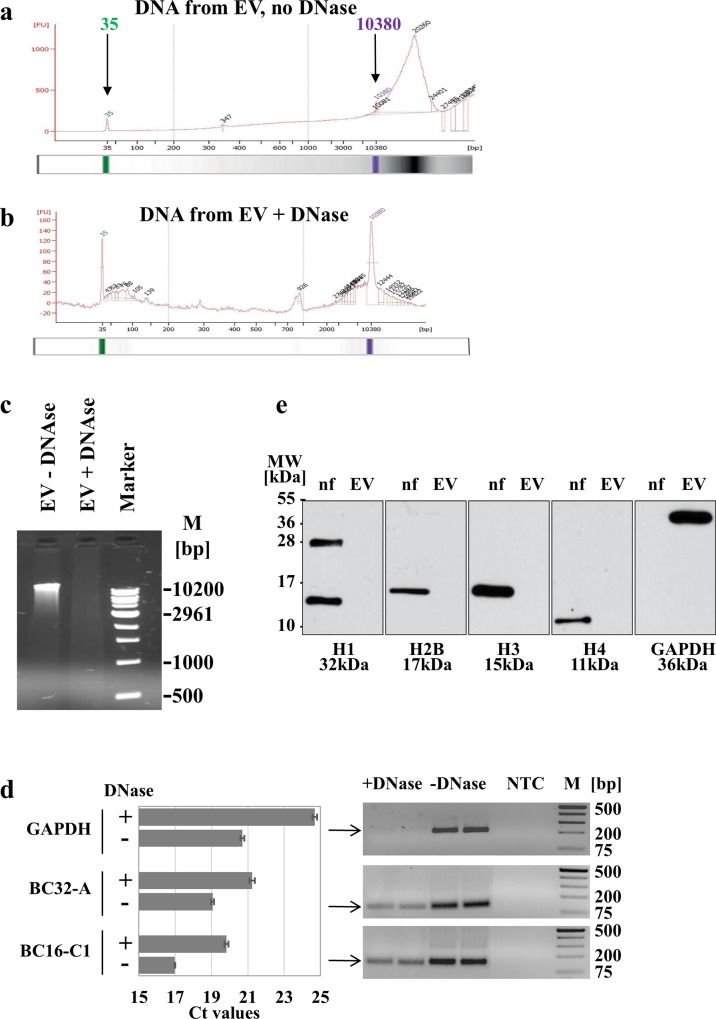
Detection of DNA in extracellular vesicles. EV were isolated from supernatants of hMSC by ultracentrifugation, divided into two parts and DNA prepared from EV without DNase treatment (a) or after DNase treatment (b). For workflow see S4 Fig. Automatically set standards of 35 (green) and 10380 bp (pink) in the Bioanalyzer indicate the lower and upper size markers. Shown are the Bioanalyzer profiles and respective gels for a representative example. (c) Ten μl from a total of 40 μl DNA sample isolated from EV without (EV no DNase) or with (EV + DNase) DNase treatment were separated on a 0.66% agarose gel. (d) To analyze the localization, DNA was isolated from unmanipulated EV (-DNase) or EV after DNase treatment (+DNase) and examined for genomic signals in quantitative PCR using primer pairs for GAPDH, BC32-A and BD16-C1 (both randomly chosen from human genome sequences). Shown are the mean Ct values ± SD of two experiments carried out in duplicates (left graph). Products of one experiment in duplicates were visualized on 1.8% agarose gels (right blots). NTC: no template control. (e) To further elucidate the composition of EV in regard to their DNA cargo, 10 μg of protein lysate were separated on 15% SDS-PAGE and analyzed for histones H1, H2B, H3, H4 and GAPDH. As positive control, a nuclear fraction (nf) of human H1299 cells was used.

Typically, DNA is purified using columns which might fragment DNA to a certain extent. Therefore we used phenol-chloroform extraction in order to minimize DNA shearing. Without DNase-treatment of EV, 25.9±14.1 ng high-molecular DNA and with DNase-treatment 8.0±6.6 ng DNA/10^6^ producer cells were isolated (n = 9), which was reflected in Bioanalyzer profiles and agarose gel (Fig 2A–2C). EV from nontransduced hMSC showed a similar DNA content (S1C Fig). DNase treatment did not destroy EV as shown in FACS analysis (S1D Fig) and sufficient DNA located inside could be extracted for next generation sequencing (S3C Fig). Additional tests of inner and outer EV-associated genomic DNA-proportions were done with qPCR showing reduction after digestion of outer DNA by 15.77, 4.5 and 7.16 fold for GAPDH, BC32-A and BC16-C1 primers, respectively (Fig 2D). The PCR products were visualized on agarose gel supporting the decrease of genomic DNA by DNase digestion (Fig 2D).

To prove that the DNA is not derived from apoptotic cells, DNA was extracted from EV of a culture with increased number of dead cells (14% Trypan-blue positive). Agarose gel analysis showed no DNA ladder typical for apoptotic cells (S2A Fig). Further, we excluded a simple co-sedimentation of DNA by ultracentrifugation (S2B–S2D Fig).

Due to their DNA cargo, we were interested in the configuration of the isolated DNA. To investigate if the EV-derived DNA was organized in nucleosomes, we prepared total protein lysate from isolated EV. After separation on a denaturing SDS-PAGE and Western blotting, we immunostained the blots for histones H1, H2B, H3 and H4. Interestingly, none of the indicated histones could be detected in our EV-derived protein sample, whereas each histone type was detected in the nuclear protein fraction (nf) used as positive control (Fig 2E). Furthermore, staining for GAPDH was used as loading control, showing a strong signal within the EV-derived protein sample, which is consistent with our previous observation (Fig 1D). Our results indicate, that the DNA isolated from EV is not organized in nucleosomes.

Next generation sequencing data of EV-derived genomic DNA from two independent EV-preparations without DNase treatment (one male and one female BM-hMSC derived EV-preparation, S3A and S3B Fig) and one EV-preparations with DNase treatment (S3C Fig) demonstrated reads that were uniformly distributed across virtually the complete human genome.

### Transfer of EV associated DNA

**Fig 3:**
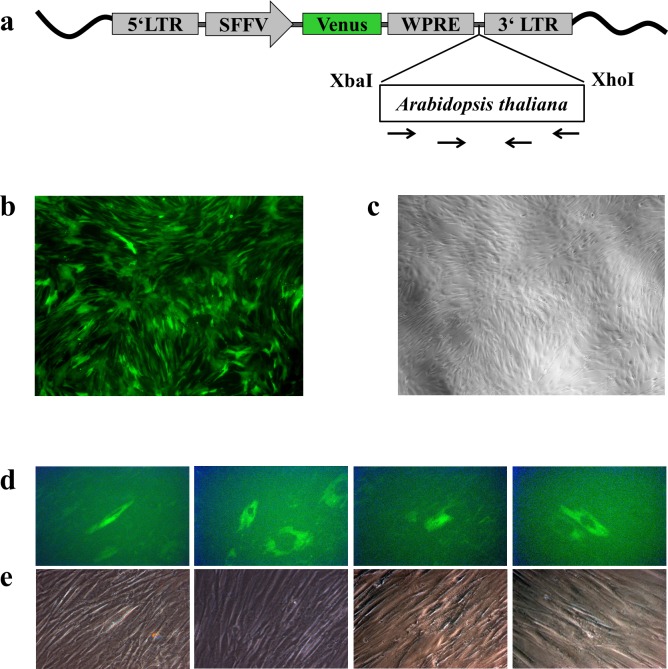
*Arabidopsis thaliana (A*.*t*.*)* virus production and transfer. (a) *A*.*t*.-DNA was cloned into the LeGO-V2-wpre plasmid vector containing Venus-fluorescence protein for detection. Primers for subsequent primary and nested *A*.*t*.-PCR shown with arrows were located within the *A*.*t*.-sequence giving rise to products of 387 bp and 106 bp respectively. (b, c) hMSC were transduced with LeGO-V2-wpre-*A*.*t*. virus supernatant. Shown is a hMSC culture 8 days after transduction (x40) detecting green cells (b) in a near confluent culture (c, phase contrast). (d, e) Recipient hMSC were incubated for 2 weeks with EV purified from hMSC-*A*.*t*. culture supernatant. Shown are Venus-positive cells (d) in the recipient culture after incubation with EV without (3 left images) or with DNase digestion (most right image) and their respective phase contrast pictures (e) (magnification x200).

The transfer of genomic DNA via EV was expected to be a rare event. Therefore, we choose a sequence of *Arabidopsis thaliana* as the DNA of interest to avoid contaminations e.g. by plasmids which have been used in the laboratory before. Sequence amplified from the *A*.*t*.-plasmid was successfully cloned into the LeGO vector backbone (Fig 3A) and transduced *A*.*t*.-hMSC were prepared for EV-production (Fig 3B and 3C).

Altogether, three separate EV preparations isolated from *A*.*t*.-hMSC with and without DNase treatment were used to investigate EV-mediated DNA transfer (see workflow S5 Fig). Recipient hMSC (3x T25 for DNase treated, 6xT25 for non-DNase treated EV) were incubated for 14 days. Within this time we assume that all coincubated EV either were endocytosed or destroyed due to their impaired integrity at 37°C [28]. After, we detected rare Venus-positive cells in cultures incubated with non-DNase digested EV (Fig 3D and 3E; first 3 images) but 1 Venus-positive cell only with DNase treated EV (Fig 3D and 3E; most right image). Irrespective of Venus-detection via fluorescence microscopy, DNA was isolated from all experimental flasks and subjected to PCR-analysis.

### Detection of *A*.*t*.-sequences

**Fig 4:**
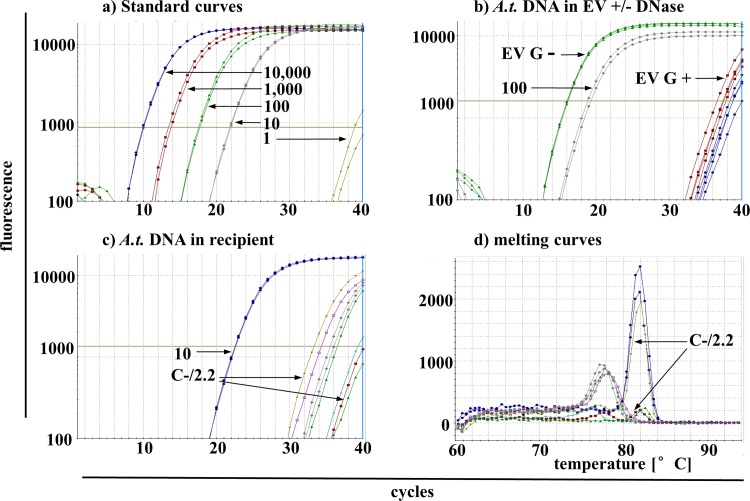
Detection of *A*.*t*.-sequences in recipient cells using SYBR Green-based qPCR. (a) Standard dilutions of *A*.*t*.-DNA in duplicates with 10.000–10 copies/PCR reaction show a linear dependency whereas 1 copy/PCR was located below the detection limit. (b) Three to four replicates of DNA isolations from EV without DNase treatment (G(-); EV from harvest G without DNAse treatment) showed high abundant *A*.*t*.-sequences with Ct = 16 whereas those with DNase treatment (G(+)) showed much lower *A*.*t*.-DNA amounts with Ct near the detection limit. As comparison, positive standard with 100 copies/PCR was plotted. (c) Several replicates of the sample C(-)16 (EV from harvest C without DNase treatment, PCR run No. 16 carried out with 1μg DNA per reaction) were detected with Ct of ≥ 33. As comparison, positive standard with 10 copies/PCR was plotted. (d) Melting temperatures (Tm) of samples in (c) show the replicates with one high and several lower peaks with the correct Tm. The blue curves correspond to the positive standard of 10 copies/PCR reaction. Two exemplary arrows for sample C(-)16 in (c) and (d) point to lime and red colored probes with high and low Tm peaks, respectively.

**Fig 5:**
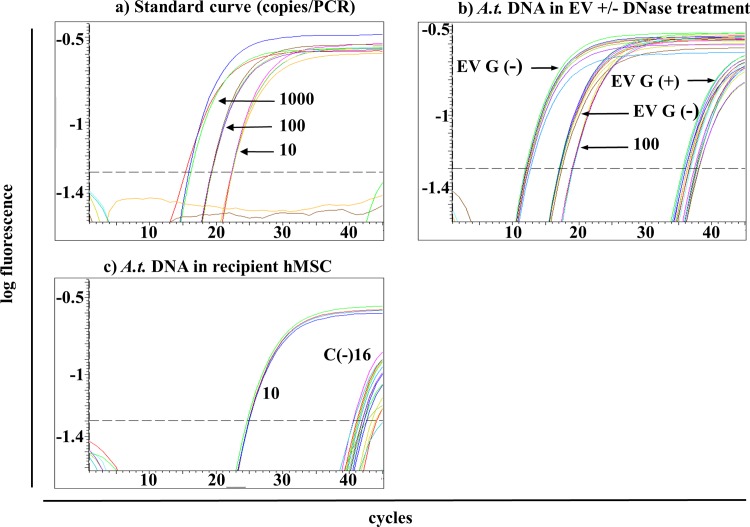
Detection of *A*.*t*.-sequences in recipient cells using TaqMan-based qPCR. (a) Standard dilutions of *A*.*t*.-DNA in quadruplicates with 1.000–10 copies/PCR show a linear dependency. (b) Eightfold replicates of two different DNA isolations from EV without (G(-)) DNase treatment showed high abundant *A*.*t*.-sequences with Ct = 13 and 18 whereas those with DNase treatment (G(+)) showed much lower *A*.*t*.-DNA amounts. As comparison, positive standard with 100 copies/PCR was plotted. (c) Several replicates of the sample C(-)16 were detected with Ct of ≥ 40. As comparison, positive standard with 10 copies/PCR was plotted. All negative controls did not give rise to signals at any time (not shown).

*A*.*t*.-transduced hMSC revealed 3.5 *A*.*t*.-copies per cell using digital droplet PCR (ddPCR). The standard curves using qPCR with 10,000 to 1 copy/PCR reaction showed reliable detection of 10–10,000 copies/PCR reaction (Figs 4A and 5A). Furthermore, two different EV preparations +/-DNase treatment (sample B and G in Fig 1G) were analyzed with ddPCR showing no *A*.*t*.-copies in sample B but abundant *A*.*t*.-sequences in sample G without and to a lesser degree also after DNase treatment (not shown). These results were corroborated in qPCR identifying high abundant *A*.*t*.-copies in sample G without DNase treatment (Ct = 17 in SYBR-based qPCR, what correlated to 100 copies/PCR reaction; Ct = 13 and 18 in TaqMan-based qPCR, what correlated to >1000 copies/PCR reaction) but low and almost at the detection limit in EV after DNase treatment (Figs 4B and 5B). Importantly, the *A*.*t*.-primers were located within the *A*.*t*.-sequence (Fig 3A) to unambiguously detect this sequence.

Applying SYBR Green-based qPCR, the analysis of DNA from EV-coincubated hMSC cultures identified potentially positive signals (Fig 4C). Particularly in sample C(-)16 (EV preparation C, see Fig 1G; (-): no DNase treatment; 16: PCR replicate No. 16) we identified positive signals which also revealed positive bands in agarose gel (not shown) and a clear product peak in the melting curve (Fig 4D). Products of this sample and additional products with low peaks but correct temperature in the melting curves were subjected to TOPO^®^ TA Cloning and sequencing. Summarised experiments showing the numbers of analyzed and therefrom positive products are listed in Table 1. Samples with a too low peak in the melting curve were not indicated as positive replicate, but single colonies subjected to sequencing. Sample C(-)16 appeared reliably positive, therefore only two colonies were tested for the correct sequence. As proof for the soundness of sequencing results, negative and positive samples have been included showing always the correct sequence in positive but no result in negative samples.

**Table 1:** Detection of *A*.*t*.-DNA in EV-recipient cells with SYBR Green-based qPCR, TOPO^®^ TA Cloning and sequencing. Shown is the total number of primary PCR replicates carried out with 1 μg DNA/PCR using complete isolated DNA of each single tissue flask, therefrom resulting nested PCR replicates, the number of positive samples in SYBR Green nested PCR, the No. of colonies which underwent sequencing and No. of colonies with the correct sequence (sequencing positive). In total, three different EV preparations were applied in this experiment. For each EV sample, 2x T25 of recipient cells were incubated with unmanipulated EV (-) and 1x T25 with EV after DNaseI (+) treatment. The nomenclature C(-)16 stands for: EV sample C without DNase treatment C(-), qPCR No. 16 carried out with 1 μg DNA per reaction.

sample name	primary PCR, No. replicates	SYBR Green nested PCR, No. replicates	No. positive replicates in nested PCR	No. colonies in TOPO Cloning	No. *A*.*t*. sequencing positive [%]
hMSC + EV sample A(-)	48	48	0	0	0
hMSC + EV sample A(+)	24	24	0	0	0
hMSC + EV sample C(-)	28	52	3	2	2 [100] C(-)16
hMSC + EV sample C(+)	15	30	1	0	0
hMSC + EV sample E(-)	38	76	2	35	4 [11.4] E(-)7
hMSC + EV sample E(+)	28	56	0	0	0 E(+)6

To increase specificity, additional TaqMan-based qPCR was performed. Investigating 80 replicates of 4 selected primary PCR, positive signals have been detected in 2 samples incubated with non-DNase digested EV. Again, the sample C(-)16 was robustly positive in several replicates (Fig 5C). This finding was verified by sequencing of several TaqMan-qPCR products showing 100% of C(-)16 and E(-)1 positive samples with the correct *A*.*t*.-sequence but none from additional negative samples E(-)7 and E(+)6 (Table 2). Products of two PCR negative samples (E(-)7 and E(+)6) as well as hMSC-negative controls were control-sequenced underlining the reliability of ExoSAP treatment (Table 2). The unique Venus-positive signal after DNase digested EV coincubation with recipient cells (Fig 3D) however could not be verified by both PCR techniques.

**Table 2:** Detection of *A*.*t*.-DNA in EV-recipient cells with TaqMan-based qPCR, ExoSAP treatment and sequencing. Shown is the total number of nested PCR replicates, the number of positive samples in TaqMan-based PCR, No. of additional negative samples included for sequencing, the summarized No. of samples which underwent sequencing and No. of samples with the correct sequence (sequencing positive). In total, PCR products of 4 primary PCR appearing positive in the SYBR Green PCR plus a negative control were applied in this experiment. The nomenclature C(-)16 stands for: EV sample C without DNase treatment C(-), qPCR No. 16 carried out with 1 μg DNA per reaction.

sample name	TaqMan nested PCR, No. replicates	No. positive replicates in TaqMan PCR	No. additional negative samples	No. ExoSAP samples	No. *A*.*t*. sequencing positive [%]
hMSC + EV sample C(-)	80	14	3	17	14 [100] C(-)16
hMSC + EV sample E(+)	80	0	3	3	0 E(+)6
hMSC + EV sample E(-)	80	7	4	11	7 [100] E(-)1
hMSC + EV sample E(-)	80	0	3	3	0 E(-)7
hMSC neg. control	60	0	3	3	0

### Inheritance of *A*.*t*. sequence

**Fig 6:**
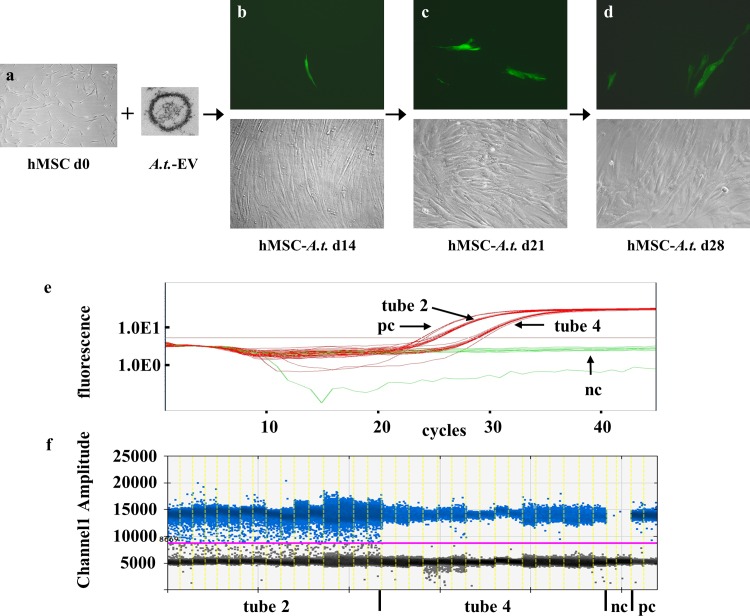
Detection of Venus-fluorescence and *A*.*t*.-sequences in recipient cells after passaging. (a) 2x10^5^ hMSC were seeded into T25, incubated overnight to reach adherence (d0) and fed with EV derived from *A*.*t*.-hMSC cultures for 2 weeks. (b) Venus-positive cells were detected in 2 of 4 flasks (d14). One flask with 7 positive cells was passaged into 4xT25 flasks. (c) 7 days later (d21), one flask contained 10 Venus-positive cells. This culture was expanded again into 4xT25. (d) Venus-positive cells at d28 were evident in 2 flasks out of 4 with 13 cells in one flask and 1 cell in the second flask. Exemplarily, one positive MSC spot with corresponding phase-contrast for each time point is shown. Magnification x 100. (e, f) DNA of the flask with 13 Venus-positive cells was pretested in nested SYBR Green-based qPCR. Out of 10 primary reaction tubes, 4 were positive in the nested qPCR tested in 8 replicates (tubes 2, 4, 7 and 8; not shown) and were retested in TaqMan-based qPCR (e) and ddPCR (f). Shown are the results for positive control (pc, 10 copies/PCR reaction; 4 replicates in TaqMan-based qPCR and 2 replicates in ddPCR), negative control (nc, untransduced hMSC; 8 replicates in TaqMan-based qPCR and 2 replicates in ddPCR), and tube 2 and 4 (16 replicates in TaqMan-based qPCR and ddPCR).

We already gained evidence that the EV-transferred *A*.*t*. sequence was passed to daughter cells (see Fig 3D, 2^nd^ and 4^th^ picture). To strengthen this, we generated new *A*.*t*.-hMSC derived EV, repeated the EV-mediated DNA transfer (Fig 6A), passaged positive cultures two times (Fig 6B–6D) and assessed the *A*.*t*. inheritance using SYBR Green-based qPCR, TaqMan-based qPCR and ddPCR (Fig 6E and 6F). Primary EV-transfer cultures again showed single Venus-positive cells: 1 in 1 flask, 7 in another and none in 2 flasks (d14, Fig 6B). The flask with 7 Venus-positive cells was expanded into 4xT25 as secondary culture. At d21 we identified 2 negative cultures, third with 1 and a fourth´ with 10 Venus-positive cells (Fig 6C). This last flask was expanded as tertiary culture presenting at d28 two negative cultures, a third flask with 1 Venus-positive cell and a fourth flask with 13 Venus-positive cells (Fig 6D). SYBR Green-based nested qPCR of the last flasks DNA identified 4 out of 10 tubes (each in 8 replicates) as positive for the *A*.*t*. signal. In tube 2, all 8 replicates were positive and in tube 4, 7 and 8 some of the 8 replicates were positive for the *A*.*t*. sequence (not shown). These four groups were retested with TaqMan-based qPCR as well as ddPCR (Fig 6E and 6F). Retesting 16 replicates of each tube in TaqMan-based qPCR (Fig 6E, results for tube 2 and 4 are shown) and 20 replicates in ddPCR (Fig 6F, 16 replicates for tube 2 and 4 are shown) verified the positivity of the samples.

## Discussion

Over the past decade the biological relevance of extracellular vesicles in intercellular communication has been established. For several years, vesicles from e.g. fibroblasts were characterized as virtually free of nuclear DNA [29, 30]. Contaminating DNA in EV repeatedly was defined as derivative from apoptotic or necrotic cells [8] which might encode some oncogenes or viral DNA [12]. Similarly, Thery *et al*. described exosomal proteins implicated in apoptosis, e.g. histones released as complexes with DNA by cells undergoing apoptosis [31]. Our data suggest that the detected DNA is not derived from apoptotic or necrotic cells, since (i) we monitored high cell survival rates during all experiments of >97%; (ii) several centrifugation steps removed necrotic cells and apoptotic bodies; (iii) no fragmented DNA typical for apoptosis was detected in agarose gel electrophoresis and bioanalyzer even in EV derived from cell cultures with high number of dead cells; (iv) DNA is not simply co-sedimented by ultracentrifugation; and (v) DNA was not organized in nucleosomes. Furthermore, our conclusion is supported by recent findings of others, also variably showing DNA in their EV preparations [32–34]. For example, chromosomal DNA sequences were identified in cardiomyocyte-derived EV [33] as well as in seminal “prostasomes” [34]. Compared to Waldenström *et al*. and Ronquist *et al*. we detected DNA not only inside the EV but significant amounts were also associated to the outer membrane what might reflect the cell source, generation and isolation of EV. Here, we showed that resulting EV generated with our protocol met all significant criteria mandatory for EV, e.g. size of <1μm and expression of typical proteins. The DNA quantities we isolated were in agreement with data from others showing 20 ng per 10^6^ cultivated HeLa cells and 52 ng per 10^6^ primary human endothelial cells [35] also underlining the importance of EV source.

Particularly tumor cells are prone to secrete considerable quantities of EV. This could be a regulated mechanism connected to their proposed role in tumor spreading as has been shown e.g. for melanoma, breast cancer and other tumors [14, 15, 21, 22]. Notably, several groups reported genomic DNA as cargo of tumor EV in a state of double-stranded DNA which represented the entire genome and reflected the mutational status of the parental tumor cells [17–19]. Our next generation sequencing results showed EV-associated DNA derived virtually from the entire genome, suggesting that horizontal transfer of the *A*.*t*.-sequence may occur irrespective of the specific integration site in the donor cells.

Enriched DNA levels in EV were described from leukemic cells in nucleosome-like complexes [20] and transposable elements in glioblastoma derived EV [16]. Particularly, the genetic information of transposable elements was available for horizontal gene transfer. Based on these findings it has been proposed to use exosomal DNA to identify mutations present in parental tumors but also for quantification and analysis of genomic mutations as biomarkers of oncogene amplification. Thus, the packaging of genomic DNA in EV from several sources could be an indication for a general property in EV formation. Considering the size of EV with 30–1000 nm, sufficient space is being available to pack RNA, proteins and high-molecular DNA. For example, the calculated internal volume of exosomes is 4.2–380 yl (10^−24^ l), so that a total cargo of ≤100 proteins and ≤10,000 net nucleotides of nucleic acid is likely [36].

To this end it became obvious that unmanipulated cells secrete quite less EV than e.g. transfected cells [37]. Haney *et al*. showed a four orders of magnitude increase of DNA and RNA incorporation in EV derived from therapeutic gene-transfected macrophages compared to those from non-modified cells. In contrast, the amount of fibroblast exosomal DNA was about 20-fold less than the amount of exosomal DNA isolated from tumor cells [17]. In our experiments, the transduced detector gene had no therapeutic function and did not change the overall characteristics of EV according to all tests we employed leading to DNA amounts comparable to unmanipulated cells. The findings that more extracellular DNA has been detected in EV derived from tumor patients could be one of the reasons why the horizontal gene transfer with EV from healthy cells appeared extremely low.

Horizontal DNA transfer has been shown previously for prokaryotes, e.g. bacteria and viruses. Though, transmission of genetic information via DNA in eukaryotes is thought to be uncommon and the DNA in chromosomes stably protected. Where does the DNA, which we reliably detected in EV, come from? Endoreduplication, for example, has been described as replication of the nuclear genome in the absence of cell division which leads to an elevated nuclear gene content [38]. It occurs when a cell exits the mitotic cell cycle in G_2_ phase and undergoes multiple S phases without entering mitosis resulting in large cells. MSC according to their size might be good candidates for endoreduplication. Although this process is rarely observed in mammals, it could increase the number of genes dedicated to tissue-specific functions [38] and to excess DNA in combination with EV, as observed in our work. We speculate that excessive DNA e.g. after endoreduplication is transported to endosomes and packaged there into newly formed EV, or alternatively freely in the cytoplasma floating DNA attaches to bilipid layers of formed EV and together with EV will be exocytosed. Indeed, using a genetic sequence from *Arabidopsis thaliana* as indicator sequence without particular function we detected DNA transfer from donor EV to recipient cells. Importantly, we exclude that the *A*.*t*.-DNA signals in recipient cells derived from remaining EV because the extended culture period and repeated medium changes ensured efficient absorption and/or complete removal of residual EV due to the limited stability at 37°C [28]. Horizontal DNA transfer, however, was an extremely rare event in our experimental setup. It has been reliably and robustly detected in 2 experiments whereas the single positive signal in cultures incubated with DNase treated EV likely has been lost during DNA preparation. Additionally, our data suggest stable integration of the detector sequence and transmission to daughter cells after several passaging. Very recently, support for DNA transfer was provided by Cai *et al*. showing that *SRY* (sex-determining region, Y) DNAs in plasma EV from coronary artery disease patients was increased. The authors concluded that EV-associated genomic DNA can be transferred to and expressed in recipient cells, thus influencing the function of the recipient cells by increasing DNA-coding mRNA and protein levels [39]. These *in vivo* results together with our *in vitro* data show that horizontal DNA transfer seems to be a rare but physiological process.

Together, the current findings suggest a coordinated impact of exosomes from both tumor cells and microenvironment to promote tumor through intercellular communication [40]. Similarly, acute and chronic myeloid leukemias at later stages were characterized by progressive fibrosis of the bone marrow showing the mutual influence in tumor formation. One mechanism in the change of cellular properties could be the horizontal DNA transfer. Although most of the functionally significant horizontal gene transfer to eukaryotes comes from bacteria [41], there are no insurmountable barriers to horizontal gene transfer, even in complex multicellular eukaryotes [42]. Currently we do not know which mechanisms caused the published differences between EV purified from different sources, though hMSC-derived EV showed abundant amounts of DNA located preferentially outside the EV bilipid layers. Several factors as e.g. cell sources, activation versus non-activation of parental cells, EV generation, purification and other parameters might explain the different outcomes in the composition of EV. This point still remains to be further elucidated.

In conclusion, we detected high-molecular DNA in association with EV derived from normal stromal cells. The high abundant DNA was transferred to recipient cells and propagated, likely in an integrated state, into the host genome. Although this was a very rare event, transfer unambiguously has been detected in several recipient samples. Together, for the first time our data indicate horizontal DNA transfer in eukaryotic cells mediated via highly purified EV, expanding the various intercellular communication pathways by an additional mechanism. This observation could be of relevance not only for leukemic gene transmissions but also for cell diversity and development.

## Materials and Methods

### Expansion of hMSC and EV generation

Bone marrow (BM) samples from healthy donors were collected after written informed consent according to the hospital’s guidelines and specifically approved by the Ethics Committee of the Medical Association Hamburg, No. PV4846. The signed documents were stored in the “Transplant coordination” of the Clinic for Stem Cell Transplantation/UKE. Human MSC were generated and expanded as described earlier [43] into Multi-Flasks (BD Biosciences, Bedford MA). For last feeding before EV generation, FCS (BioWhittaker, Apen, Germany) was EV-depleted by overnight centrifugation with 120,000 xg at 4°C for ≥18 h. Majority of EV thus was removed from FCS [44, 45]. EV were generated in medium supplemented with 0.5% EV-depleted (analogous to FCS) bovine serum albumin (BSA, Sigma-Aldrich, Taufkirchen, Germany) + 2 mM Glutamax (Gibco, Karlsruhe, Germany) for 48 h. EV were harvested from the supernatant at 4°C using a modified centrifugation protocol published by Thery *et al*. [44]. Centrifugation was carried out for 20 min at 2,000 xg to deplete cells and debris and 12,000 xg for 20 min to deplete apoptotic bodies and large vesicles. The EV were pelleted for 70 min at 100,000 xg using Beckman Coulter ultracentrifuge equipped with swinging bucket rotor, washed in PBS, resuspended in small volumes α-MEM (Minimum Essential Medium) + 25mM HEPES (both Gibco) and stored at -80°C. Cells in the Multi-Flasks were “recycled” after the first supernatant harvest by feeding with expansion medium supplemented with EV-depleted FCS for 24 h, thereafter starting next round of EV-generation for 48 h without trypsinization. This schedule was repeated up to 6 times. Thereafter, hMSC were lifted with Trypsin-EDTA (Gibco) and counted using Trypan-Blue (Sigma-Aldrich) for live-dead discrimination.

### Characterization of EV

#### Electron microscopy

For ultrastructural analyses, purified EV were encapsulated in cellulose capillary microtubes (Microdyn, Wuppertal, Germany) and processed as described by Hauber *et al*. [46]. Briefly, the microtubes (200 μm diameter) were filled with the EV suspensions by capillary action and mechanically sealed at both ends with a blunt side of a dermal curette. The samples were fixed with 2.5% glutaraldehyde (EMS, Hatfield, PA, USA) in PBS, 1% uranyl acetate (Merck, Darmstadt, Germany) and 1% OsO_4_ (Carl Roth, Karlsruhe, Germany) in ddH_2_O for 30 min each, followed by dehydration in a graded series of ethanol (all chemicals: Sigma). For ultrathin sectioning, the microtubes were embedded in EPON resin (Agar Scientific, Stansted, UK). Sections were poststained with 2% uranyl acetate and lead citrate (Leica Microsystems, Wetzlar, Germany). Electron micrographs were acquired with a Multiscan 794 camera (Gatan, Pleasanton, CA, USA) attached to a Philips CM 120 TEM (Philips, Eindhoven, Netherlands) operated at 80 kV.

For the *in situ* analysis of EV budding cells were grown on Ibidi dishes (ibidi GmbH, Planegg, Germany) and monitored by phase contrast microscopy in a Nikon Biostation IM. Preselected this way, cells were fixed with 2.5% glutaraldehyde in PBS, 1% uranyl acetate and 1% OsO_4_ in ddH_2_O for 30 min each, and dehydrated in graded series of ethanol. The whole cell monolayer was embedded in EPON resin as a thin film. Punched-out pieces of the EPON film containing the cells were glued onto EPON blocks and sectioned. Ultrathin sections were poststained with 2% uranyl acetate and lead citrate. Electron micrographs were acquired with a Gatan Multiscan 794 camera attached to a Philips CM 120 TEM operated at 80 kV.

#### Western blotting

Human BM-MSC derived EV were lysed in Lämmli buffer, the protein concentration determined with Lowry Assay (Bio-Rad Laboratories GmbH, München, Germany) in a NanoDrop 1000 spectrophotometer (PEQLAB Biotechnologie, Erlangen, Germany) and 10 μg of protein lysate analyzed by 10% SDS-PAGE electrophoresis and Western transfer onto PVDF membrane using standard procedures. Primary antibodies against CD9, CD63, CD81 and Hsp70 were used at 1:1000 and horse reddish peroxidase-coupled (HRP) secondary goat anti-rabbit at 1:20,000 concentrations (EXOAB, System Biosciences, CA, USA). Detection was done with SuperSignal West Pico Chemoluminescence Reagent (Thermo Fisher Scientific, Waltham, MA, USA).

#### Quantification

Quantification of EV was carried out with the FACSAriaIIIu (BD Biosciences, Heidelberg, Germany). Submicron Bead Calibration Kit (0.2/0.5/0.76 μm; Bangs Laboratories, Fishers, IN, USA) and Flow Cytometry Size Calibration Kit (1/2 μm; Thermo Fisher Scientific, Waltham, MA, USA) were used to define the counting gate up to 1 μm. Sure Count Particle Standard 3 μm (Bangs Laboratories) were placed within the plot area for subsequent quantification of EV. Additionally, 0.1 μm filtered PBS and Count Particles diluted in 0.1 μm filtered PBS were evaluated for contamination with submicron particles.

To evaluate the impact of “swarm detection” of small particles in FACS [27] we additionally employed NanoSight measurements for EV concentration and size (NanoSight LM 14, Malvern Instruments GmbH, Herrenberg, Germany). Data were acquired in 10 repeats for 10 sec each per EV sample (n = 6).

#### Determination of DNA in EV

DNA was extracted using Phenol-Chloroform extraction from (i) purified EV, or (ii) purified EV after treatment with 100U DNaseI (Thermo Fisher) to eliminate external DNA (see workflow S4 Fig). Dried DNA pellet was resuspended in 40 μl ddH_2_O. One μl of purified DNA was analyzed using High Sensitivity DNA Kit on the Agilent 2100 Bioanalyzer (Agilent Technologies, Waldbronn, Germany), 10μl out of 40μl DNA of (i) and (ii) were separated on 0.66% agarose gel.

To exclude that the EV-associated DNA was derived from apoptotic cells, EV were purified from a culture with increased number of dead cells (14% Trypan-blue positive), the DNA extracted thereof and analysed on agarose gel. To further exclude a simple co-sedimentation of DNA with EV by ultracentrifugation, EV were purified in parallel using (a) ultracentrifugation, (b) Exo-spin kit (Cell Guidance System Ltd., Cambridge, UK), and (c) exosome isolation kit (101Bio, Palo Alto, CA, USA).

Additionally, to determine the portion of DNA located in- or outside with EV, genomic templates were analyzed with 30 ng DNA purified from unmanipulated EV (-DNase) and DNase-treated EV (+DNase) with 3 primer pairs. Two primer pairs were designed to be randomly located within the genome, a third primer pair was recognizing genomic GAPDH with the following sequences: BC16-C1 forward (FW) 5‘-GCTGGAGTGCAATGGTGTTA-3‘ and reverse (RV) 5‘-AAAATTAGCTGGGCATGGTG-3‘ (126bp, located on chromosome 9), BC32-A FW 5‘- AAAATTACGTGGGCATGGTG-3‘ and RV 5‘-AGAGTGCAGTGGCCTGATCT-3‘ (124bp, located on chromosome 8), GAPDH FW 5‘-CACTTGATTTTGGAGGGATGTCG-3‘ and RV 5‘-ACCAGGGCTGCTTTTAACTCTGG-3‘ (200bp, located on chromosome 12) (all primers: Eurofins Genomics, Ebersberg, Germany). Two experiments of quantitative PCR in duplicates were carried out for 25 cycles in a MX3000Pro Thermocycler (Agilent, Santa Clara, CA, USA) with SYBR® Premix *Ex Taq*™II (TaKaRa Bio, Otsu, Japan). The products of were visualized on 1.8% agarose gel.

Furthermore BM-hMSC derived EV protein extracts were prepared in RIPA lysis buffer containing 1% (v/v) PMSF, 0.1% (v/v) aprotinin, 1μg/ml leupeptin, 1μg/ml pepstatin A, and 1mM DTT (Sigma). Alternatively, cells were fractionated as described by Lee *et al*. [47] before protein extracts of the nuclei were prepared in RIPA buffer. Protein lysates were separated by 15% SDS-PAGE and immobilized by Western transfer onto PVDF membranes using standard procedures. Primary antibodies against histones H1, H4 (both Santa Cruz, Dallas, TX, USA), H2B and H3 (both Abcam, Cambridge, UK), and GAPDH (Cell Signaling Technology, Danvers, MA, USA) were used as recommended by the manufacturer. Immunostaining with corresponding secondary antibodies and visualization were done as described above.

#### Next generation sequencing (NGS) of EV-derived DNA

The extracted DNA of 2 separate EV-preparations (one male and one female BM-hMSC donor) without and 1 after DNase treatment was fragmented using the Bioruptor (Diagenode, Boston, MA) with 7 cycles of 30 sec on/off followed by library preparation with the NEXTflex ChIP-Seq Kit (option 2; Bioo Scientific, Austin, TX, USA) according to manufacturer’s recommendations. Fragment length distribution of the libraries was analyzed on a BioAnalyzer 2100 High Sensitivity Chip (Agilent Technologies). Diluted libraries (2nM) were multiplex-sequenced on the Illumina (San Diego, CA, USA) HiSeq 2500 instrument (2x 125 bp paired end run, 100 million reads/sample).

The Burrows-Wheeler Aligner (BWA mem) [48] was employed to align sequence reads to the human reference assembly (UCSC HG19). Based on these alignments, normalized coverage was computed over intervals of 500 kb using FREEC [49]. Coverage data were plotted with R [50].

### Production and titration of viral supernatant

The plant *Arabidopsis thaliana* (*A*.*t*.*)* DNA sequence was PCR-amplified out of the plasmid GI-Gal4 DBD (Addgene, Cambridge, MA) using standard procedures. The primers FW 5´-ATATCTCGAGGGGCAACTGATGGAA-3´ and RV 5´-TTGATCTAG AAGAGCAAGCTGTGAGCT-3´ gave rise to a 413 bp fragment. The obtained fragment was ligated via the beforehand introduced XbaI and XhoI cloning sites into LeGO vector backbone [51] giving rise to LeGO-V2-wpre-*A*.*t*. vector containing Venus-fluorescence protein for detection. Production of viral supernatants and titration were performed as published by Weber *et al*. [52]. Tropism of the replication incompetent virus for human cells was defined by using the gibbon ape leukemia virus (GALV) envelope.

### Transduction of primary hMSC

Human BM-derived MSC were seeded into 6-well plates (Greiner, Frickenhausen, Germany) 12 h before transduction. Adherent cells were transduced at multiplicities of infection (MOI) of 10 as described [52]. Two days later, Venus-positive cells (*A*.*t*.-hMSC) were sorted, expanded and used for EV generation as described above. Non transduced hMSC were carried along as controls for all subsequent experiments.

### EV-transfer to recipient cells

Recipient human BM-hMSC (2.5-5x10^4^) unrelated to hMSC used for *A*.*t*.-transduction were seeded into T25-flasks (Greiner). Single, not pooled EV preparations derived from three individual *A*.*t*.-hMSC cultures were divided into two parts. One part was left untreated, the second part underwent DNase treatment (see workflow S5 Fig). EV were added to recipient hMSC in excess of EV derived from 600 *A*.*t*.-hMSC (EV without DNase treatment) or 1,000 (EV with DNase treatment) to 1 hMSC and left for 2 weeks with culture medium change twice a week. Cultures were controlled regularly with an IX-81 Olympus inverted fluorescence microscope (Olympus, Hamburg, Germany) and morphology and Venus-fluorescence documented. After 14 days, genomic DNA was extracted from each single flask using QIAamp® DNA Mini Kit (Qiagen, Hilden, Germany).

### Digital droplet PCR (ddPCR)

Isolated DNA from *A*.*t*.-hMSC MOI 10 (QIAamp® DNA Mini Kit, Qiagen) was used for vector copy number determinations by ddPCR. In a duplex reaction, a vector-specific fragment (primers FP-dPCR-fw, FP-dPCR-rv and the FAM- labelled FP-probe as described in [50]) and a house keeping amplicon (human erythropoietin receptor, primers FW 5‘-CTGCCAGCTTTGAGTACACTA-3‘, RV 5‘-GAGATGCCAGAGTCAGATACCACAA-3‘, probe 5‘-HEX-ACCCCAGCTCCCAGCTCTTGCGT-BHQ1-3‘) were simultaneously amplified. In total, 200 ng of genomic DNA were used as template for 40 cycles of PCR according to the manufacturer´s protocol. Droplets were generated and analyzed using the QX100 system (BioRad, München, Germany).

The ddPCR was also carried out for EV without or with excessive DNAse treatment with 40U/5x10^7^ EV (Thermo Fisher) to detect the *A*.*t*.-sequence in distinct EV preparations. For these reactions, the primers and probe from TaqMan-based qPCR specific for the *A*.*t*. sequence were used.

### Detection of *A*.*t*.-DNA

DNA from *A*.*t*.-hMSC MOI 10 spiked with DNA from untransduced hMSC was used to establish a standard curve of *A*.*t*.-copy numbers. For a single PCR reaction, 1 μg in 5 μl template containing 100,000 down to 1 copy were prepared and used to determine the *A*.*t*. copy number in the investigated DNA.

To detect *A*.*t*.-DNA in recipient cells, primary *A*.*t*.-PCR was carried out with 1 μg extracted DNA/reaction for 25 cycles using Q5® High Fidelity DNA Polymerase (New England Biolabs, Ipswich, MA, USA) with the primers FW 5‘-TGTGAGCTTCCCAGTTTAAACA-3‘ and RV 5‘-GAGGGGCAACTGATGGAATG-3‘ (386 bp) located within the *A*.*t*.-sequence. Altogether, total isolated DNA from each individual experimental flask was subjected to primary PCR. The product was diluted 1:100 with ddH_2_O and 5 μl were used as template for a nested qPCR with SYBR® Green and primers FW 5‘-TTCCGTTCTTCTCTGTTGTTGG-3‘ and RV 5‘-GCACACGTACGTGCCTTAAG-3‘ for 40 cycles (106bp). Each qPCR run was accomplished by a melting curve. The PCR products were electrophoretically separated on 3% agarose gels. Positive signals were cut out, extracted with QIAquick Gel Extraction kit (Qiagen) and cloned using TOPO^®^ TA Cloning^®^ kit (Thermo Fisher). After *Blue-White-Screening* on Agar plates, Sanger sequencing from selected colonies was done at Seqlab-Microsynth (Göttingen, Germany) using the primer 5‘- GTAAAACGACGGCCAG-3‘. Additionally, products with the correct melting temperature in the final curve but without a positive signal in agarose gel were subjected to TOPO^®^ TA Cloning and sequencing too.

As an alternative with enhanced *A*.*t*.-DNA detection specificity, TaqMan qPCR (Peltier Thermal Cycler Chromo 4, Bio-Rad Laboratories, München, Germany) using primers FW 5‘- GTTGAAGAATCGATAGGACGGACTA-3‘, RV 5‘-CCATACCCATCAAAGTAACTC CAA-3‘ and the probe 5‘-FAM -TCATTCCGTTCTTCTCTGTTGTTGGCAGT- BHQ1-3‘ to detect low-abundant *A*.*t*.-DNA amounts after the primary PCR was performed. Potentially positive products were treated with ExoSAP (Affymetrix, High Wycombe, UK) and sequenced. In all experiments, DNA purified from untransduced MSC-EV was used as negative control. Importantly, no-template controls from the primary and the nested PCR were included to carefully detect unspecific contamination.

### Inheritance of transferred *A*.*t*. DNA

EV-transfer to recipient hMSC was repeated with a new batch of Venus-*A*.*t*.-positive hMSC derived EV, the uptake and expression of Venus-fluorescence documented after 2 weeks and positive cultures passaged twice by trypsinization with regular documentation of Venus-positive cells. Then the DNA was isolated, pretested with nested SYBR Green-based qPCR and positive tubes probed for *A*.*t*.-sequences by TaqMan-based qPCR and ddPCR as described above.

## Supporting Information

S1 Fig Characterization of EV derived from untransduced hMSC. (a) Quantification of total EV amounts per harvest (16 samples from five individual donors 4–8) revealed similar amounts among all harvests. When total EV amounts were blotted against the protein content of each EV harvest, interindividual differences in protein cargo but reproducibility within one donor culture after repeated EV harvests were observed. (b) Purified EV in gate P1 were quantified using counting beads excluding the particles contained in filtered PBS as shown specifically in Fig 1E and 1F. (c) DNA purified from untransduced hMSC-derived EV with or without DNase treatment showed similar quantities of DNA cargo compared to *A*.*t*.-transduced hMSC-derived EV (n = 5). (d) EV were not destroyed by DNase treatment as shown by quantification in flow cytometry.
(PDF)

S2 Fig EV-associated DNA is not derived from apoptotic cells and not co-sedimented by ultracentrifugation. (a) EV were isolated from supernatants of hMSC culture with decreased viability of 86%. DNA was purified by phenol-chloroform extraction and indicated amounts separated on a 0.66% agarose gel. Even under this conditions, the EV-associated DNA did not show the typical fragmentation in form of DNA ladder. (b-d) EV were isolated from equal amounts of supernatants of hMSC cultures with ultracentrifugation (b), Exo-spin kit (c), or exosome isolation kit (d; for this isolation 1/6 of the supernatant amount was used due to the limited capacity of the columns), DNA was isolated and the DNA-Bioanalyzer profiles were recorded. Automatically set standards of 35 (green) and 10380 bp (pink) in the Bioanalyzer indicate the lower and upper size markers. Shown are the Bioanalyzer profiles and respective gels (left). Nanosight quantifications (right) show the amounts of a 1:1000 dilution and size of isolated EV. In (d), no quantification was possible due to low EV amounts. The results show high-molecular DNA irrespective of the isolation method.
(PDF)

S3 Fig Next generation sequencing of genomic DNA isolated from BM-hMSC derived EV. EV were generated from BM-hMSC supernatant, left untreated or digested with DNase as described and DNA purified from concentrated EV. Sequence reads were aligned to the human reference assembly UCSC HG19 and normalized coverage computed over intervals of 500kb. Shown are the data for undigested EV derived from a female bone marrow donor (a), a male donor (b) and DNase treated EV derived from a female bone marrow donor (c).
(PDF)

S4 Fig Schematic workflow for detection and evaluation of genomic DNA in EV. Purified EV from supernatant of expanded bone marrow-derived hMSC were devided into 2 parts. First part was left untreated before DNA isolation via Phenol/Chloroform extraction, second part was DNase treated followed by Phenol/Chloroform-extraction. The dried DNA was resuspended in 40 μl aqua dest. One μl of each isolated DNA was examined on a Bioanalyzer. Ten μl of EV -/+ DNase treatment were separated on a 0.66% agarose gel.
(PDF)

S5 Fig Schematic workflow for investigating DNA transfer by EV. Human MSC of three individual donors were transduced with a lentiviral *Arabidopsis thaliana*-vector giving rise to *A*.*t*.-hMSC. Cells were expanded until three Multiflasks with 5 layers reached confluence (❶). The last medium change before EV production was carried out with EV-depleted FCS containing medium. No residual lentiviral particles in free or cell-bound form should be present due to lentiviral lifetime. EV-production was done for 48 hours in 0.5% EV-depleted BSA containing medium followed by a 24 hour recovering period in EV-depleted FCS containing medium. This cycle was repeated up to 6 times to harvest EV without trypsinization of the culture (❷). EV producer cells (❸) were subjected to digital droplet PCR (ddPCR) to establish *A*.*t*. copy numbers for subsequent PCR examination, as has been done for produced EV (❹) to detect *A*.*t*.-DNA associated with EV. Next, EV harvest of 3 individual *A*.*t*.-hMSC donor-cultures was divided into 2 parts. One part was treated with DNase (+), the second part left untreated (-) and new unrelated recipient cells were incubated with the EV (❺). For each individual EV preparation, 2 flasks (I and II) were incubated with untreated EV and one flask (I) with DNase-treated EV. The recipient cells were fed biweekly for two weeks (❻). Within this time all coincubated EV either were endocytosed or destroyed due to their short survival at 37°C. After this period, DNA was isolated from each individual flask and subjected to primary PCR (❼). The PCR products were diluted 1:100 and examined in a nested SYBR Green-based PCR for the *A*.*t*. sequence (❽). Potentially positive products according to Ct values and melting curves were separated on agarose gel, extracted from gel, TOPO cloned and subjected to blue-white screening. Positive colonies where picked and sequenced to verify the *A*.*t*. sequence (❾). Nested PCR of all positive and several negative and control samples was repeated with highly sensitive TaqMan-based PCR (❿). The PCR products were treated with the ExoSAP kit and sequenced to verify the *A*.*t*. sequence (⓫).
(PDF)
