# Supplementary material for: Indication of Horizontal DNA Gene Transfer by Extracellular Vesicles
Source: PLoS One. 2016 Sep 29;11(9):e0163665. doi: 10.1371/journal.pone.0163665 (PMC5042424; doi:10.1371/journal.pone.0163665)
Supplement: S1 Fig — (a) Quantification of total EV amounts per harvest (16 samples from five individual donors 4–8) revealed similar amounts among all harvests. When total EV amounts were blotted against the protein content of each EV harvest, interindividual differences in protein cargo but reproducibility within one donor culture after repeated EV harvests were observed. (b) Purified EV in gate P1 were quantified using counting beads excluding the particles contained in filtered PBS as shown specifically in Fig 1E and 1F. (c) DNA purified from untransduced hMSC-derived EV with or without DNase treatment showed similar quantities of DNA cargo compared to A.t.-transduced hMSC-derived EV (n = 5). (d) EV were not destroyed by DNase treatment as shown by quantification in flow cytometry. (PDF) [file pone.0163665.s001.pdf]

a

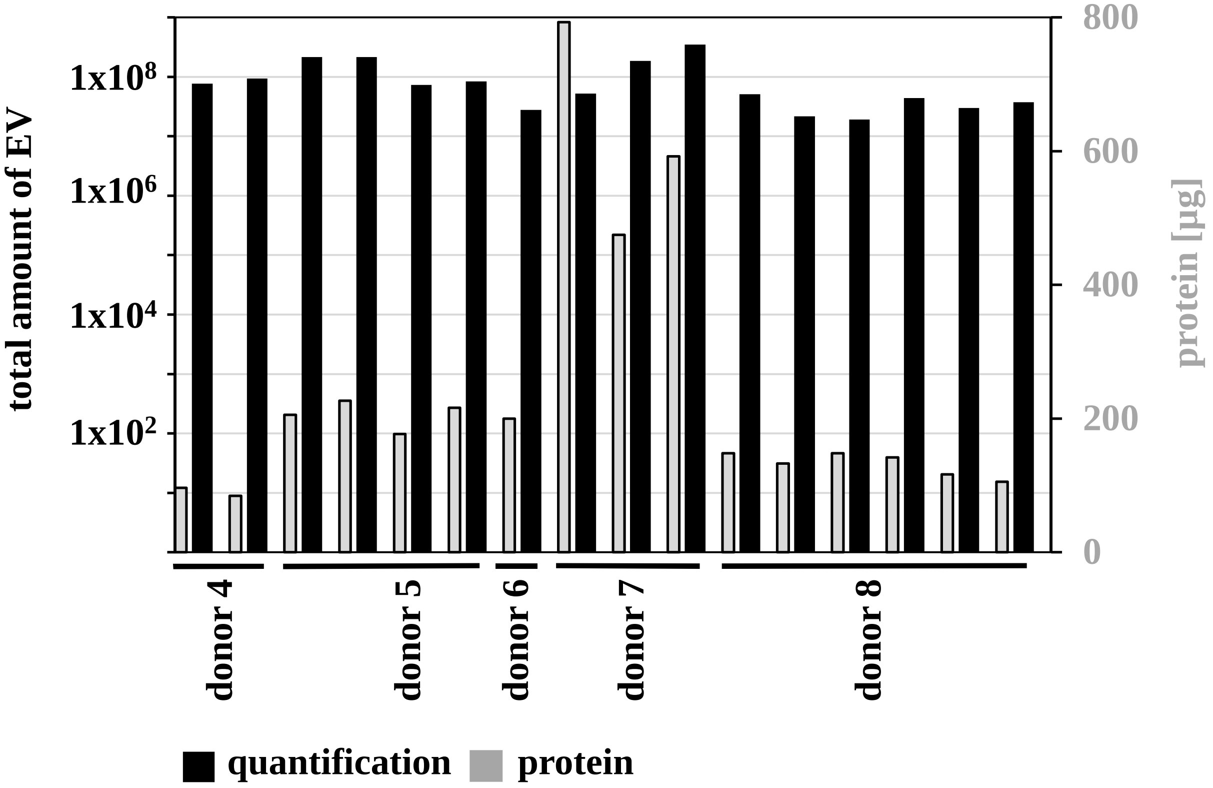

total amount of EV
1x10^8
1x10^6
1x10^4
1x10^2
800
600
400
200
0
protein [µg]
donor 4
donor 5
donor 6
donor 7
donor 8
quantification
protein

b EV quantification

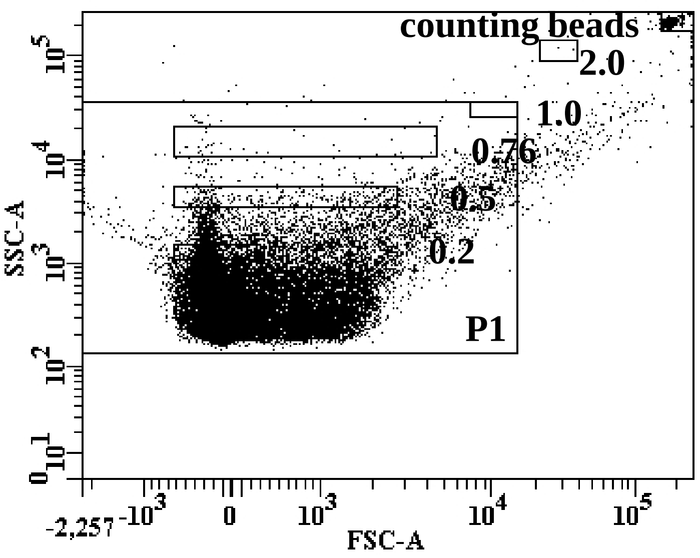

c

EV without DNase digestion [ng/10^6 producer cells]	EV with DNase digestion [ng/10^6 producer cells]
24.38 ± 9.04	5.09 ± 3.95

d

EV without DNase

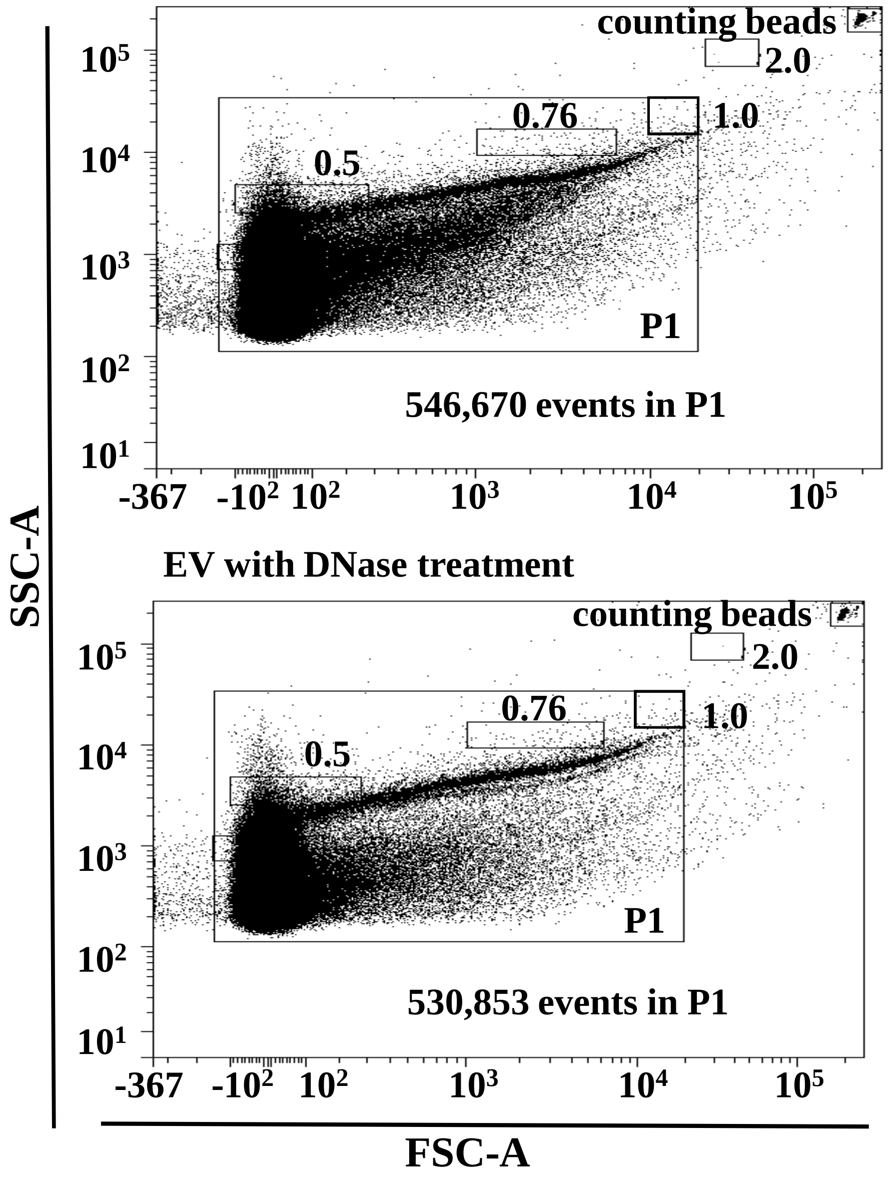

S1 Fig.
