# Supplementary figures and images for: Indication of Horizontal DNA Gene Transfer by Extracellular Vesicles
Source: PLoS One. 2016 Sep 29;11(9):e0163665. doi: 10.1371/journal.pone.0163665 (PMC5042424; doi:10.1371/journal.pone.0163665)

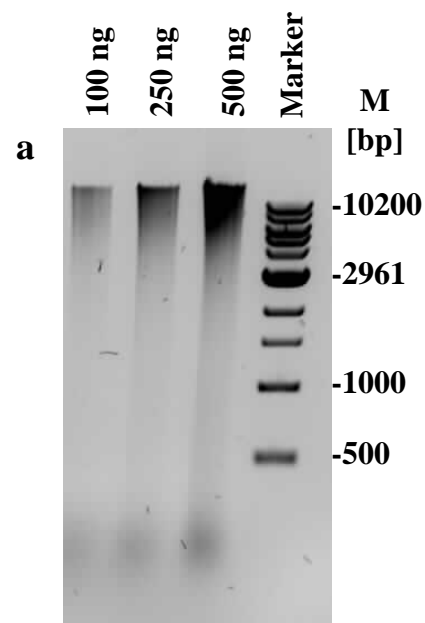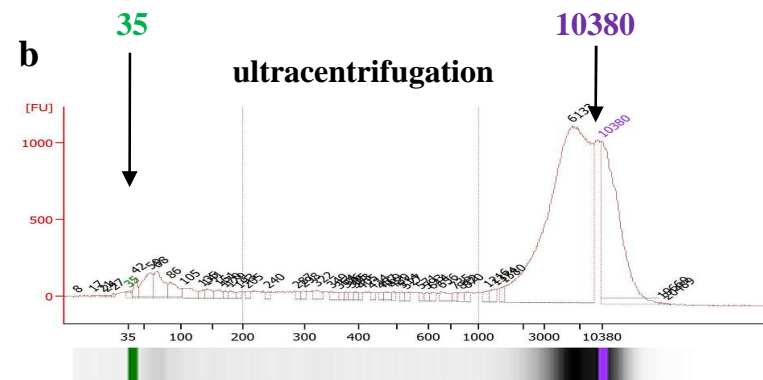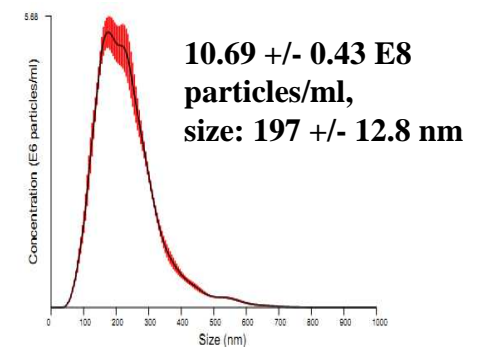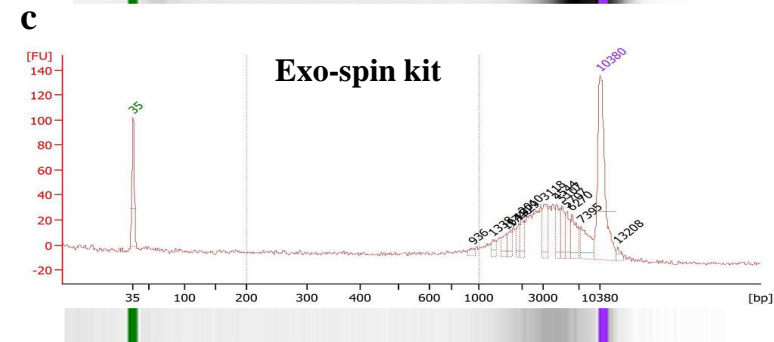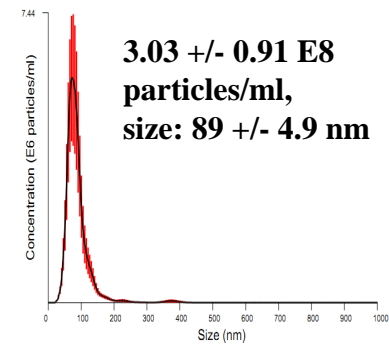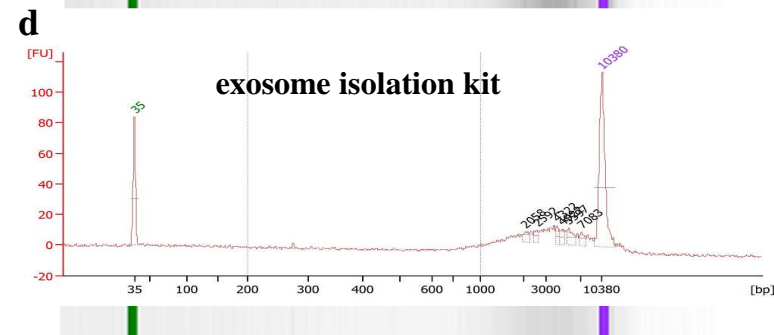

S2 Fig

Supplement: S2 Fig — (a) EV were isolated from supernatants of hMSC culture with decreased viability of 86%. DNA was purified by phenol-chloroform extraction and indicated amounts separated on a 0.66% agarose gel. Even under this conditions, the EV-associated DNA did not show the typical fragmentation in form of DNA ladder. (b-d) EV were isolated from equal amounts of supernatants of hMSC cultures with ultracentrifugation (b), Exo-spin kit (c), or exosome isolation kit (d; for this isolation 1/6 of the supernatant amount was used due to the limited capacity of the columns), DNA was isolated and the DNA-Bioanalyzer profiles were recorded. Automatically set standards of 35 (green) and 10380 bp (pink) in the Bioanalyzer indicate the lower and upper size markers. Shown are the Bioanalyzer profiles and respective gels (left). Nanosight quantifications (right) show the amounts of a 1:1000 dilution and size of isolated EV. In (d), no quantification was possible due to low EV amounts. The results show high-molecular DNA irrespective of the isolation method. (PDF) [file pone.0163665.s002.pdf]

**a**

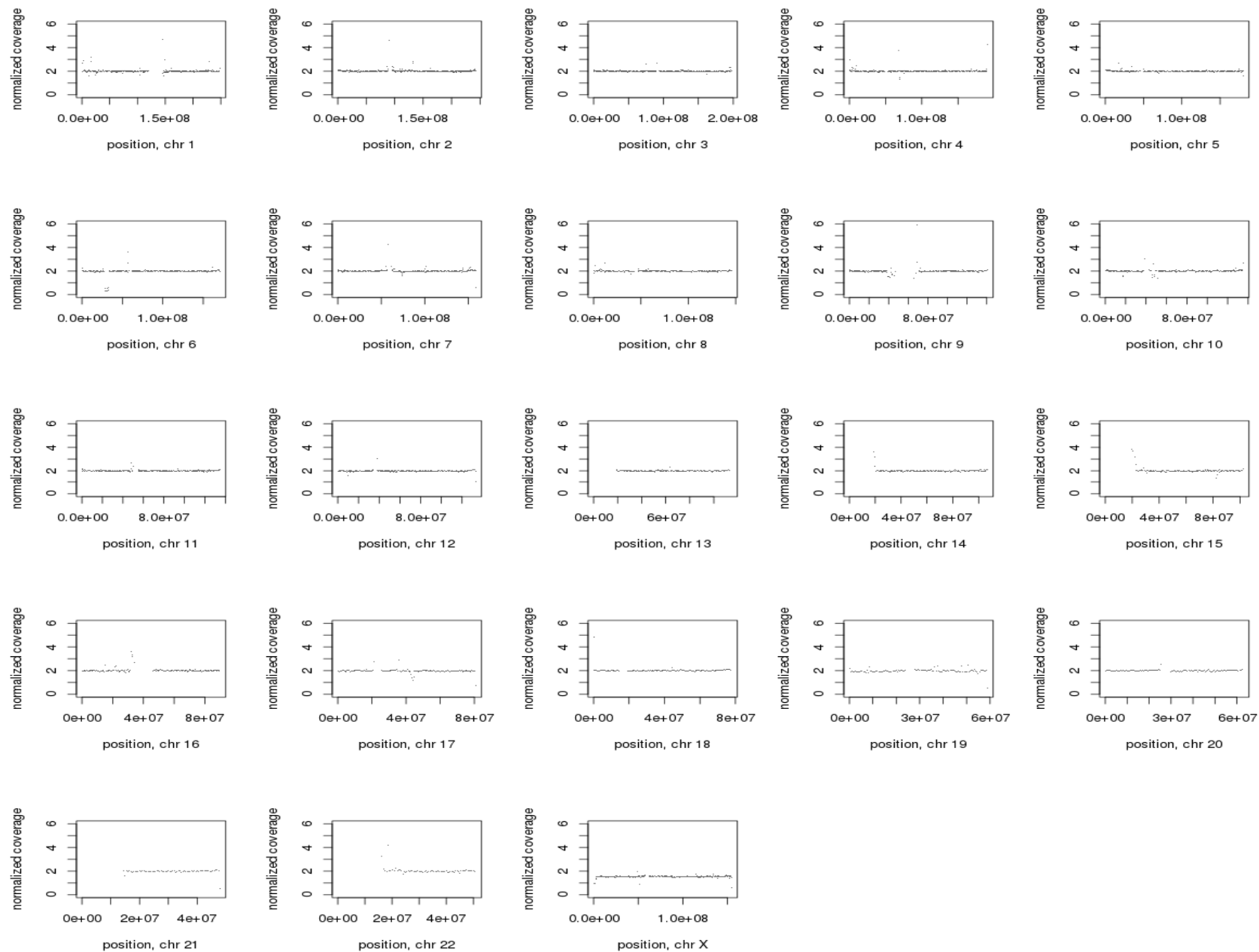

**b**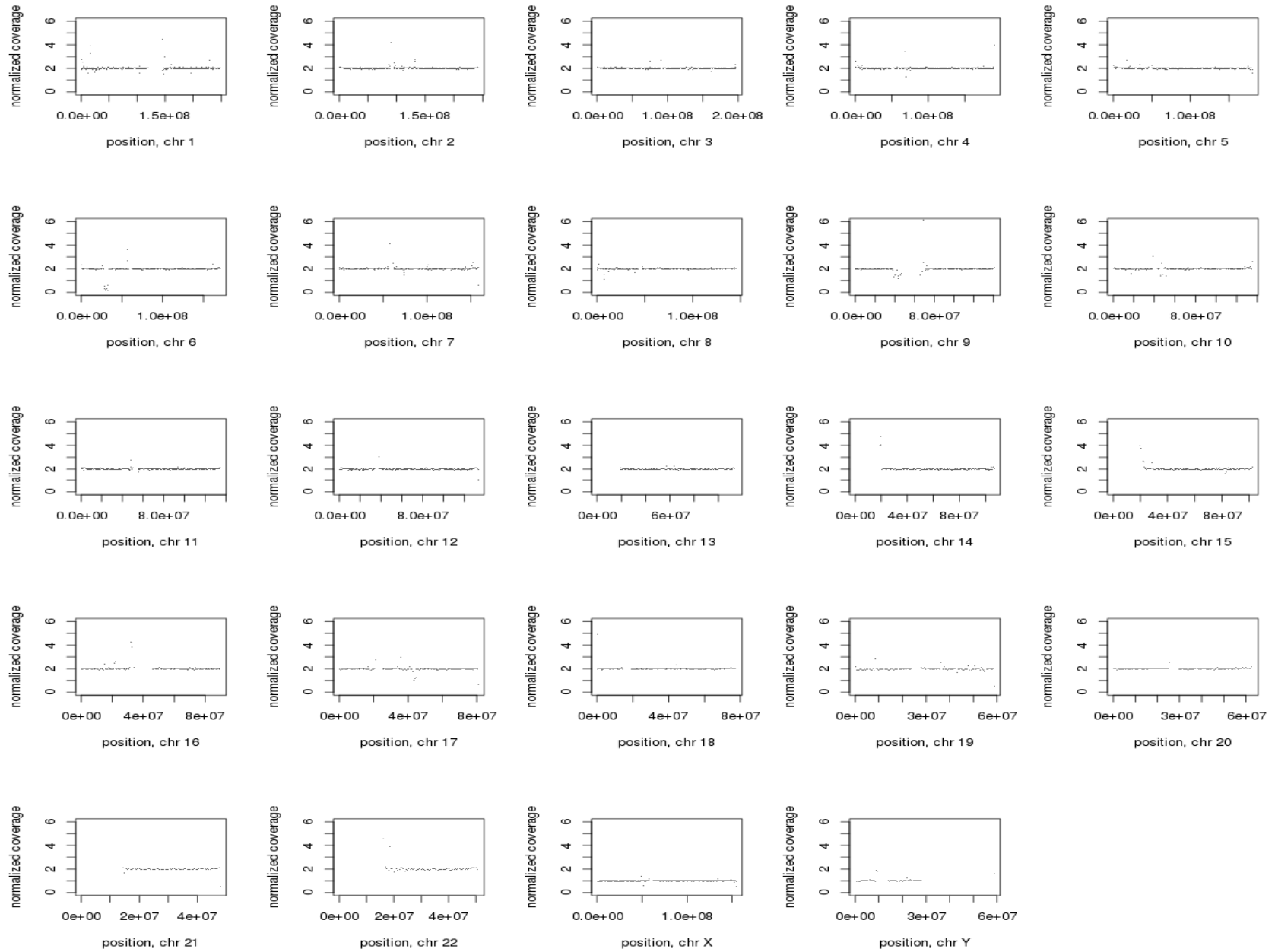

**c**

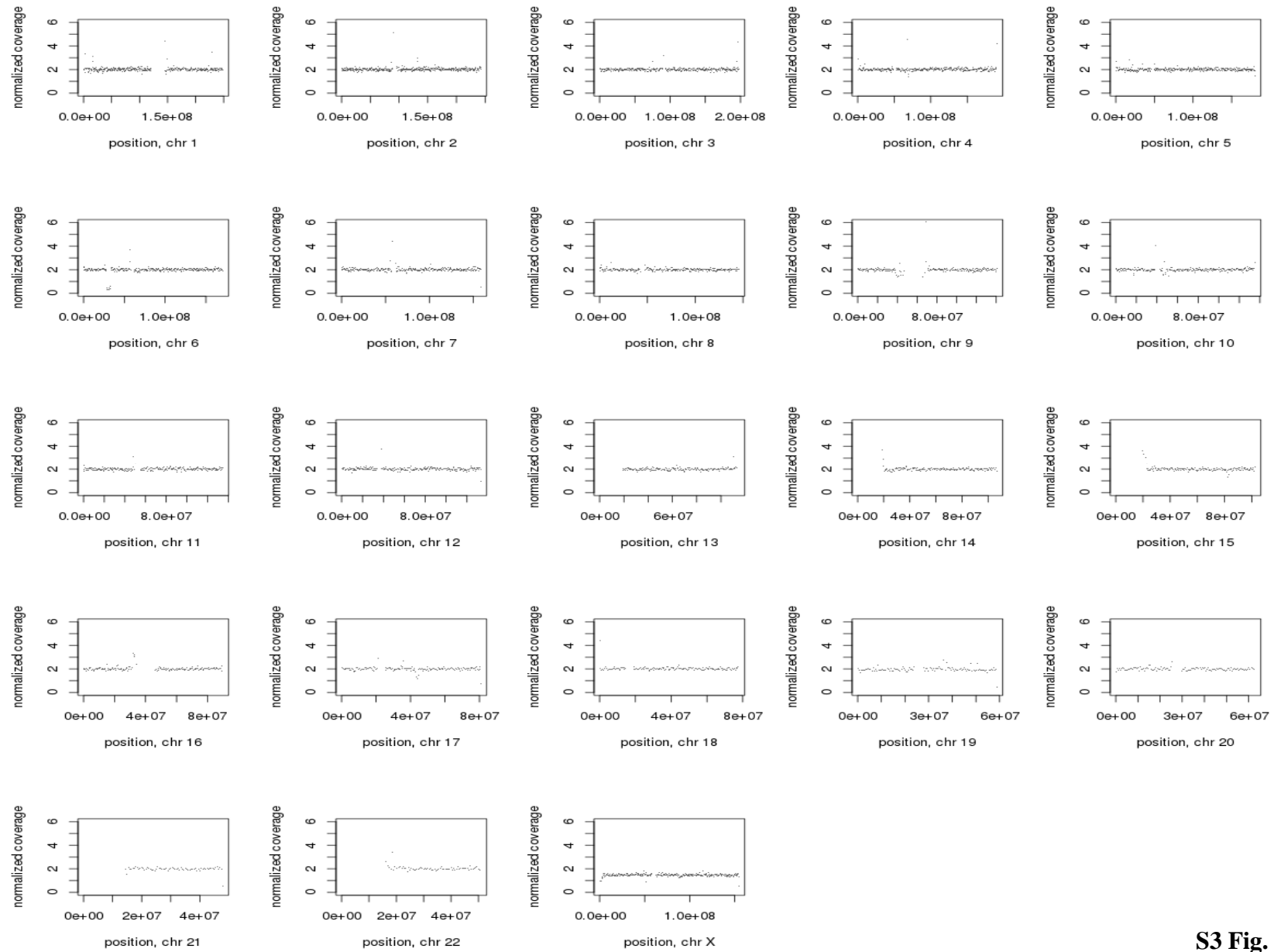

**S3 Fig.**

Supplement: S3 Fig — EV were generated from BM-hMSC supernatant, left untreated or digested with DNase as described and DNA purified from concentrated EV. Sequence reads were aligned to the human reference assembly UCSC HG19 and normalized coverage computed over intervals of 500kb. Shown are the data for undigested EV derived from a female bone marrow donor (a), a male donor (b) and DNase treated EV derived from a female bone marrow donor (c). (PDF) [file pone.0163665.s003.pdf]

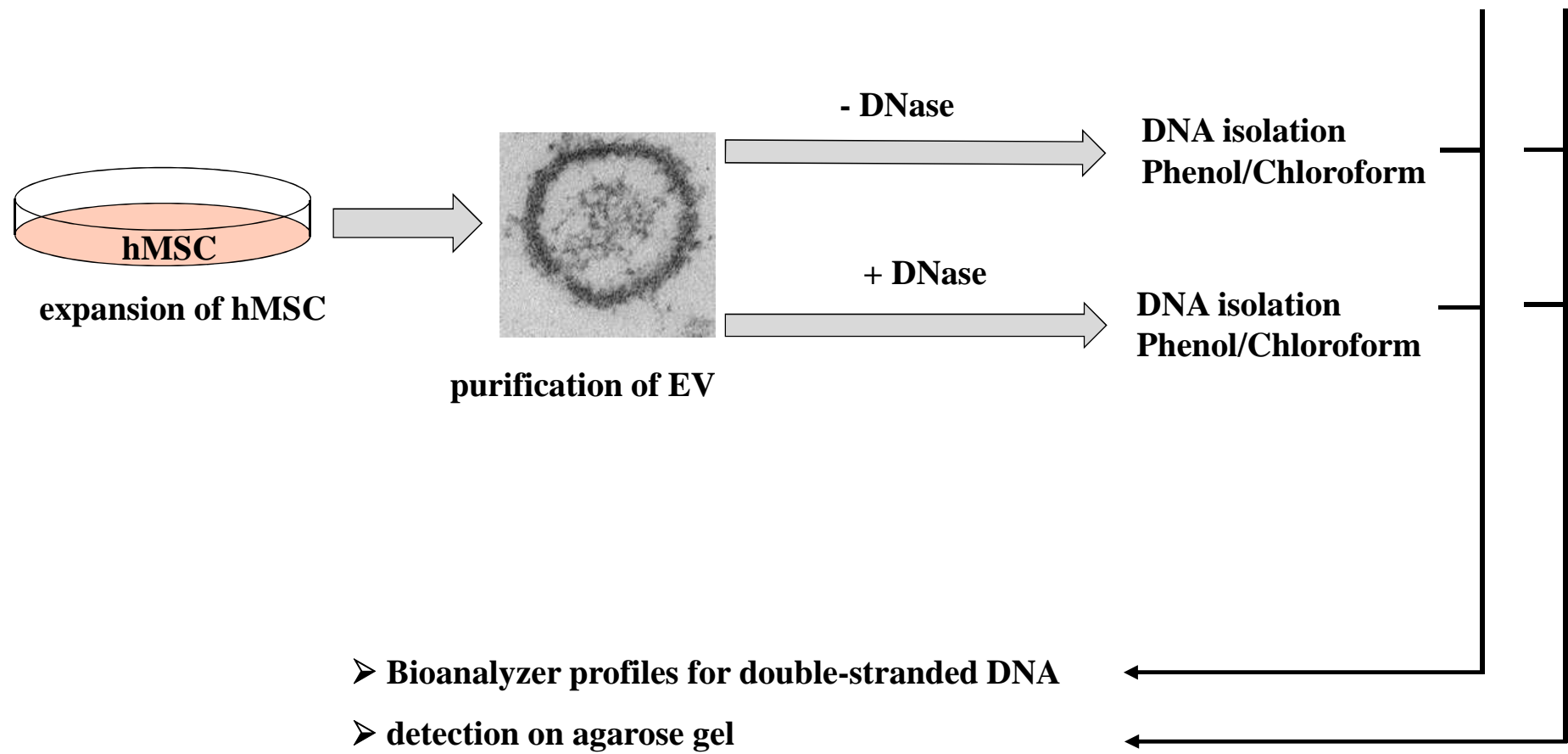

**S4 Fig.**

Supplement: S4 Fig — Purified EV from supernatant of expanded bone marrow-derived hMSC were devided into 2 parts. First part was left untreated before DNA isolation via Phenol/Chloroform extraction, second part was DNase treated followed by Phenol/Chloroform-extraction. The dried DNA was resuspended in 40 μl aqua dest. One μl of each isolated DNA was examined on a Bioanalyzer. Ten μl of EV -/+ DNase treatment were separated on a 0.66% agarose gel. (PDF) [file pone.0163665.s004.pdf]

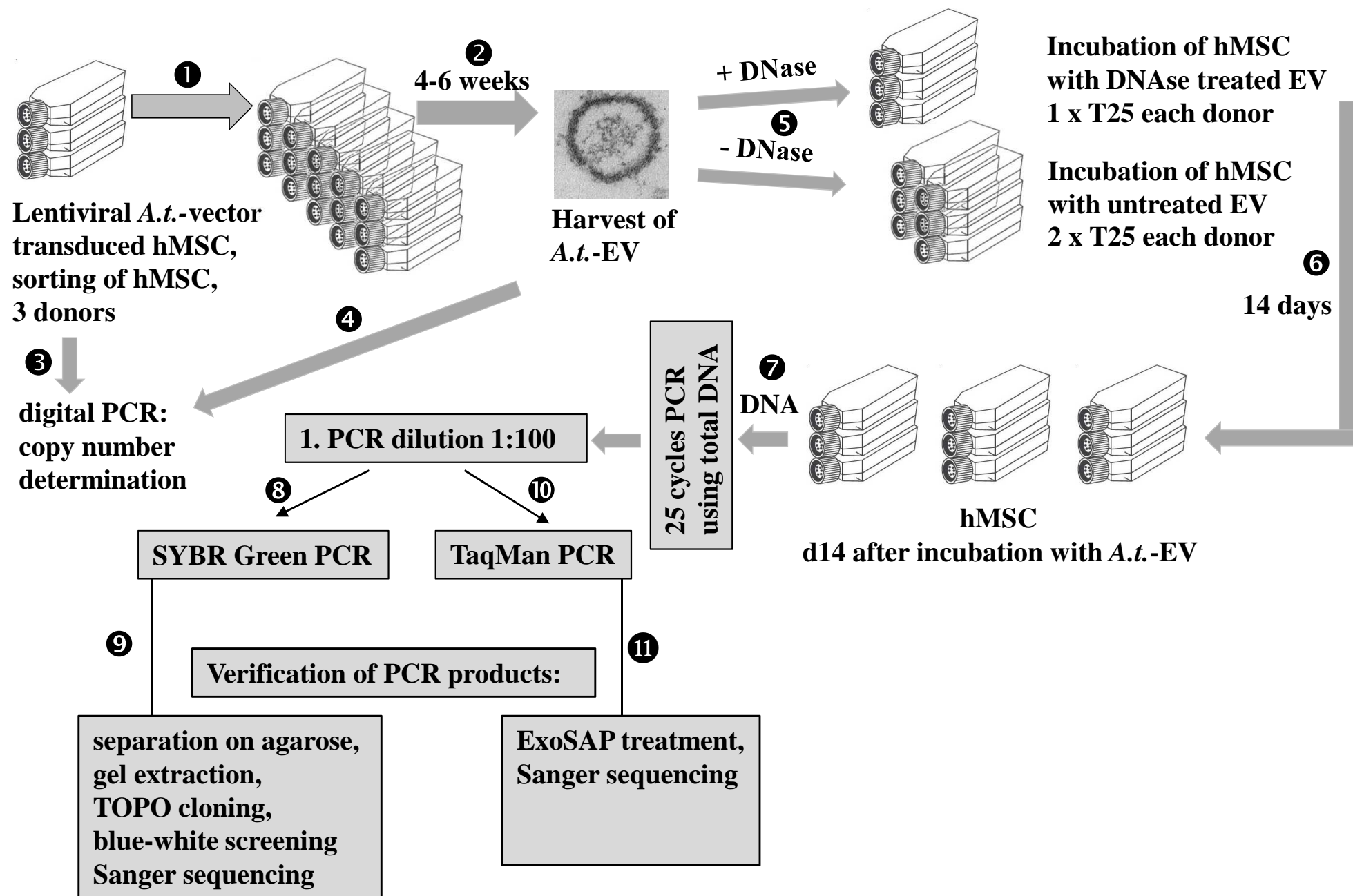

S5 Fig.

Supplement: S5 Fig — Human MSC of three individual donors were transduced with a lentiviral Arabidopsis thaliana-vector giving rise to A.t.-hMSC. Cells were expanded until three Multiflasks with 5 layers reached confluence (❶). The last medium change before EV production was carried out with EV-depleted FCS containing medium. No residual lentiviral particles in free or cell-bound form should be present due to lentiviral lifetime. EV-production was done for 48 hours in 0.5% EV-depleted BSA containing medium followed by a 24 hour recovering period in EV-depleted FCS containing medium. This cycle was repeated up to 6 times to harvest EV without trypsinization of the culture (❷). EV producer cells (❸) were subjected to digital droplet PCR (ddPCR) to establish A.t. copy numbers for subsequent PCR examination, as has been done for produced EV (❹) to detect A.t.-DNA associated with EV. Next, EV harvest of 3 individual A.t.-hMSC donor-cultures was divided into 2 parts. One part was treated with DNase (+), the second part left untreated (-) and new unrelated recipient cells were incubated with the EV (❺). For each individual EV preparation, 2 flasks (I and II) were incubated with untreated EV and one flask (I) with DNase-treated EV. The recipient cells were fed biweekly for two weeks (❻). Within this time all coincubated EV either were endocytosed or destroyed due to their short survival at 37°C. After this period, DNA was isolated from each individual flask and subjected to primary PCR (❼). The PCR products were diluted 1:100 and examined in a nested SYBR Green-based PCR for the A.t. sequence (❽). Potentially positive products according to Ct values and melting curves were separated on agarose gel, extracted from gel, TOPO cloned and subjected to blue-white screening. Positive colonies where picked and sequenced to verify the A.t. sequence (❾). Nested PCR of all positive and several negative and control samples was repeated with highly sensitive TaqMan-based PCR (❿). The PCR products were treat [file pone.0163665.s005.pdf]
